# Interference from semantically distracting sounds in action scene search

**DOI:** 10.3758/s13414-025-03023-8

**Published:** 2025-02-06

**Authors:** Tomoki Maezawa, Miho Kiyosawa, Jun I. Kawahara

**Affiliations:** 1 University of Tsukuba, 1-1-1 Tennodai, Tsukuba, Ibaraki 305-8577 Japan; 2 Hokkaido University, Sapporo, Japan

**Keywords:** Crossmodal, Attention, Audiovisual, Auditory enhancement, Visual search

## Abstract

Research on visual searching has highlighted the role of crossmodal interactions between semantically congruent visual and auditory stimuli. Typically, such sounds facilitate performance. Conversely, semantically incongruent sounds may impair visual search efficiency for action scenes, though consensus has yet to be reached. This study investigated whether interference effects occur within the action-scene search paradigm. Participants performed a search task involving four simultaneously presented video stimuli, accompanied by one of three sound conditions: sound congruent with the target, congruent with a distractor, or a control sound. Auditory interference was observed, though it was relatively weak and varied across conditions rather than being simply present or absent. The observed variability in interference effects may align with the established view that observers typically ignore semantic distractor information in goal-directed searches, except in cases where the strength of target designation is compromised. These findings offer insights into the complex interplay between auditory and visual stimuli in action scene searches, suggesting that these underlying mechanisms may also apply to other paradigms, such as those involving conventional real object searches.

## Introduction

Auditory stimuli can significantly influence the selective processing of visual stimuli at the source location. Sounds can involuntarily capture listeners’ spatial attention, enhancing object detection and localization across sensory modalities (Driver & Spence, 1999; Eimer & Driver, 2001; Feng et al., 2014; McDonald et al., 2003, 2013, 2014; Maddox et al., 2015; Mossbridge et al., 2011; Spence & Driver, 1997; Störmer, 2019; van den Brink et al., 2014). While the advantage of crossmodal spatial cueing is well-documented, the impact of semantic congruence or incongruence between auditory and visual stimuli has received less attention. However, in real-world situations, sounds characteristic of particular objects provide valuable cues about their visual appearance, such as a bark indicating a dog. Consequently, recent research has increasingly focused on how such auditory cues facilitate or interfere with visual searches in cluttered environments (Iordanescu et al., 2008, 2010, 2011; Kvasova et al., 2019).

The mechanisms underlying these semantic cueing effects, although not fully understood, likely involve audiovisual integration (Iordanescu et al., 2008, 2010, 2011). In this context, high-level semantic information from auditory cues can coactivate visual representations of corresponding objects, enhancing sensitivity to their low-level features (Knoeferle et al., 2016). Object-identification studies offer strong evidence for this audiovisual interaction; visual items are identified more accurately and rapidly when paired with congruent sounds compared with incongruent or irrelevant sounds (Chen & Spence, 2010, 2011; Cox & Hong, 2015; Hein et al., 2007; Mastroberardino et al., 2015; Molholm et al., 2004; Spilcke-Liss et al., 2019; Zweig et al., 2015). Identification research further indicates that visual representations may be activated within 300 ms of an auditory signal (Chen & Spence, 2010). Thus, characteristic sounds that convey congruent information alone are sufficient to facilitate the search for relevant object features, similar to spatial cueing effects (Iordanescu et al., 2008, 2010, 2011; Kvasova et al., 2019).

Considering the framework of audiovisual semantic integration, it is not surprising that semantically incongruent information can also affect visual search (see Chen & Spence, 2010; Laurienti et al., 2004; Spilcke-Liss et al., 2019; Suied et al., 2009). For instance, when auditory cues represent target-irrelevant objects, they may inadvertently enhance the representations of these distractors, potentially directing attention away from the target. However, this assumption is opposed by evidence that minimizes the impact of distractor-congruent sounds (Iordanescu et al., 2008, 2010, 2011; Kvasova et al., 2019; see also Cox & Hong, 2015; Laurienti et al., 2004). Studies suggest that, unlike in simple identification tasks, auditory and visual distractors can sometimes be ignored during a search (Iordanescu et al., 2008, 2010, 2011; Kvasova et al., 2019). This may be due to the goal-directed attentional set that prioritizes current tasks, even when auditory cues enhance sensitivity to distractors (Chen & Zelinsky, 2006). Such goal-driven searching may create an asymmetric pattern in search times: While target-congruent sounds typically facilitate search, distractor-congruent sounds consistently fail to cause interference (Iordanescu et al., 2008).

**Fig. 1:**
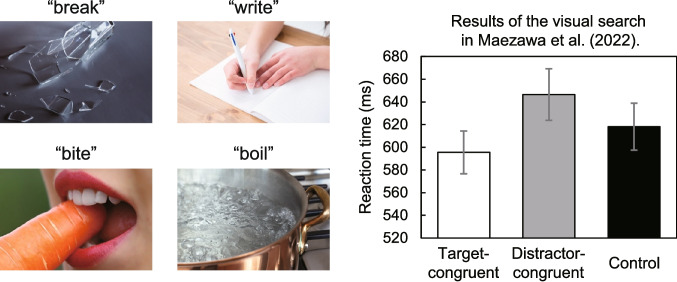
Illustrative examples from an action-scene search study (Maezawa et al., 2022). The graph presents the reaction times for “target-congruent,” “distractor-congruent,” and “control” sound conditions averaged across six experiments. The still images are partially adapted from the cited study

The present study investigated whether the lack of interference is a consistent characteristic of visual search (Iordanescu et al., 2008). The stability of this asymmetric search pattern remains unclear (see Chen & Spence, 2010), as previous research has demonstrated both facilitation and interference in visual search tasks (Maezawa et al., 2022). This study explores real object-search paradigms (Iordanescu et al., 2008) by incorporating an action scene search. In this paradigm, participants were asked to localize colored images that corresponded to predefined target actions (e.g., “breaking,” “writing,” and “biting”; see Fig. 1) rather than the real objects (e.g., “dog,” “key,” and “piano”; see Iordanescu et al., 2008). Notably, despite the minor change in stimuli, consistent interference was observed in five of six experiments using various control sounds, contrasting with the asymmetric patterns documented in previous studies (Iordanescu et al., 2008, 2010, 2011; Kvasova et al., 2019). Identifying whether interference is an inherent aspect of visual search remains complex, particularly due to variation in experimental paradigms.

To further explore whether interference is a consistent feature of visual search, we replicated the auditory interference found in action-scene searches (Maezawa et al., 2022). One possible factor contributing to the complexity of this phenomenon may be the use of static images, which are uncommon in daily experiences. These static images, depicting brief snapshots of action scenes, do not adequately capture the dynamic qualities typically associated with such actions. Considering that these stimuli are rarely encountered in real-world settings, participants may need to engage in interpretive processes to assess their alignment with the target action. While this interpretive demand remains speculative, it is absent in searches for well-defined tangible objects (Iordanescu et al., 2008, 2010, 2011; Kvasova et al., 2019). Consequently, the reliance on artificial stimuli complicates direct comparisons with traditional real-object search paradigms, further obscuring the nature of the observed interference.

Accordingly, this study examined action-scene searches by substituting static images with dynamic images (i.e., short video clips) depicting action scenes. These stimuli may provide more comprehensive information about the actions, resembling real-life observations. We examined whether this substitution would produce similar outcomes, particularly whether it would elicit interference from distractor-congruent sounds. To this end, three experiments were conducted in the first half of the study, where participants localized a target video clip within a visual array. In the second half, two control experiments compared interference patterns between searches for static and dynamic images.

## Experiments 1–3

Experiments 1–3 replicated the effects of semantically congruent and incongruent sounds on visual searches for dynamic action scenes (i.e., examining auditory facilitation and interference). The visual search tasks were adapted from those designed for real object searches (Iordanescu et al., 2008) and action-scene searches using static images (Maezawa et al., 2022). Participants were instructed to localize a visual target after its name was presented. The search array consisted of four simultaneous video clips, one representing the target scene and the remaining three depicting distractor scenes. During the visual search, one of three characteristic sounds was played: a target-congruent sound, distractor-congruent sound, or control sound. The control condition varied across experiments: “unrelated sound” in Experiment 1, “white noise” in Experiment 2, and “no sound” in Experiment 3, as outlined below. Differences in reaction times relative to controls were used to identify interference and facilitation effects.

### Methods

#### Participants

In total, 75 naïve undergraduate and graduate students from Hokkaido University participated in the study (41 men and 34 women; mean age = 19.17 years, range: 18–24 years), with equal distribution across the three experiments. Participants received either monetary compensation or course credit. The sample size was consistent with a previous action-scene search study (Maezawa et al., 2022), in which a post hoc power analysis using G*Power 3 (Faul et al., 2007, 2009) indicated a power of 0.969 with *N* = 25, α = 0.05, η_p_^2^ = 0.277 (*f* = 0.620), and ε = 1.00 for detecting effects of sound presentations on search performance. All participants had normal color vision, normal hearing ability, and normal or corrected-to-normal visual acuity.

#### Apparatus and stimuli

The study utilized 20 dynamic action scenes representing events commonly observed in daily life, such as “break,” “write,” “close,” “stir-fry,” “cut,” “bite,” “burn,” “turn” (turning pages), “sweep,” “tear” (tearing paper), “slap,” “giggle,” “boil,” “fly,” “run,” “cry,” “type” (typing letters), “sneeze,” “knock,” and “pour” (available on the Open Science Framework at https://osf.io/ab7uw/). Each action was represented by a silent video clip (25 frames per s, original length of 1,000 ms) and a monophonic audio clip (ranging from 592 to 1,555 ms in original length; mean length = 1,367 ± 274 ms) independent of the videos. The duration of audio clips varied depending on the nature of the sound. Video and audio stimuli were collected separately through web searches, ensuring that they corresponded to the same action-related events, such as the sound of glass shattering for “break” and the sound of a pen rubbing against paper for “write.” Thus, the video and audio clips were semantically consistent but not perfectly temporally synchronized.

**Fig. 2:**
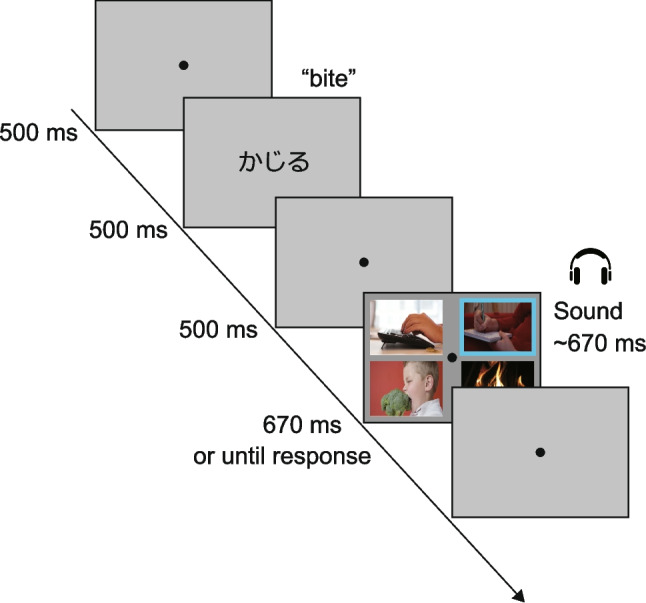
Representative sequence of stimulus presentation. The search display illustrates a case where the target scene (“bite”) is positioned in the lower left quadrant. Three nontarget items are positioned in the other quadrants. The distractor-congruent sound corresponds semantically to the nontarget item positioned diagonally opposite the target (cyan frame). Note that the color frame is for illustrative purposes only and was not visible in the actual experiment. (Color figure online)

The visual stimuli were presented on an LCD monitor (100 Hz; 1,920 × 1,080 pixels; XL2411T; BenQ) against a gray background, whereas auditory stimuli were delivered via headphones (15–28,000 Hz; K361; AKG) at a comfortable volume. Stimulus presentation was controlled using MATLAB (Version R2019a; The MathWorks) with Psychophysics Toolbox 3.0 extensions (Brainard, 1997; Kleiner et al., 2007; Pelli, 1997). Participants maintained a viewing distance of approximately 57 cm. Each video clip subtended a visual angle of 17.57° (width) × 9.94° (height) on the monitor. The search array displayed four video clips simultaneously, each positioned within one of four quadrants (Fig. 2). The center of each video clip was positioned at an eccentric angle of 12.39° from the center of the monitor, with horizontal and vertical separations of 20.80° and 13.22° between clip centers, respectively.

#### Procedure

Each trial of the action-scene search task began with a fixation point (0.33° in diameter) presented at the screen center for 500 ms. Next, the name of the target action (e.g., “bite”) that participants needed to locate was presented visually in Japanese for 500 ms, followed by a blank screen with the fixation point for an additional 500 ms. Subsequently, the search display appeared, consisting of one target video clip and three nontarget video clips positioned in one of four quadrants (lower left, upper left, lower right, or upper right). Upon display onset, all four videos played simultaneously. One of the 20 audio clips was selected to play synchronously with the video playback. Both video and audio clips ended either 670 ms after onset or when the participant responded, after which the display transitioned to a blank screen.

During each trial, one of three sound conditions (i.e., target-congruent, distractor-congruent, or control) was randomly assigned. In the target-congruent condition, the sound matched the target video clip semantically. In the distractor-congruent condition, the sound was semantically congruent with the nontarget video clip (i.e., distractor) positioned diagonally opposite the target across the fixation point (see Fig. 2). This placement minimized any spatial proximity effects between the target and distractor, as close positioning could inadvertently facilitate target detection by the distractor-congruent sound, complicating accurate measurement of interference effects (Iordanescu et al., 2008). The control condition sound varied across experiments: In Experiment 1, an unrelated sound, semantically unrelated to search items, was selected from one of the other 20 audio clips. In Experiment 2, we used Gaussian white noise (670 ms), whereas in Experiment 3, no sound was played during the search display.

Participants were instructed to localize the target action scene rapidly and accurately by pressing one of four numeric keys (1, 4, 2, or 5) on a standard keypad. The keypad was arranged in a square layout corresponding to the quadrants of the search display. Participants responded using the index and middle fingers of both hands: the left forefinger pressed the “1” key, the left middle finger the “4” key, the right forefinger the “2” key, and the right middle finger the “5” key.

A one-way within-subjects factorial design was employed, featuring three levels corresponding to the sound conditions. The visual search task consisted of four blocks, each comprising 60 trials (20 trials per condition), resulting in a total of 240 trials. Within each block, the 20 audio clips were randomly assigned to trials across the sound conditions, with each clip presented once per condition. Similarly, the 20 video clips were randomly assigned as target stimuli for the search task once per trial within each condition. The remaining three nontarget videos in the search display were selected without replacement from the remaining 19 videos (excluding the target video), ensuring balanced presentation across trials. As mentioned previously, in the target-congruent condition, the target video was semantically congruent with the accompanying audio, whereas in the distractor-congruent and control conditions, the target was semantically incongruent with the sounds. The visual search task commenced with an instructional display, followed by 10 practice trials before the experimental trials began.

### Results

Reaction times were recorded from the onset of the search array, with analyses focused on correct responses. Error trials, including those where participants failed to respond within 30 s, and outliers, defined as reaction times more than 3 standard deviations above or below the mean, were excluded from the reaction time analyses. To examine the effects of sound presentations on visual search performance, data were analyzed using a one-way repeated-measures analysis of variance (ANOVA), followed by post hoc pairwise *t* tests (two-sided) with Holm correction. No violations of specificity assumption (Greenhouse–Geisser) were found in the collected data. All statistical analyses were conducted using JASP software (Version 0.17.1; JASP Team, 2023).

#### Experiment 1 (unrelated sound)

**Fig. 3:**
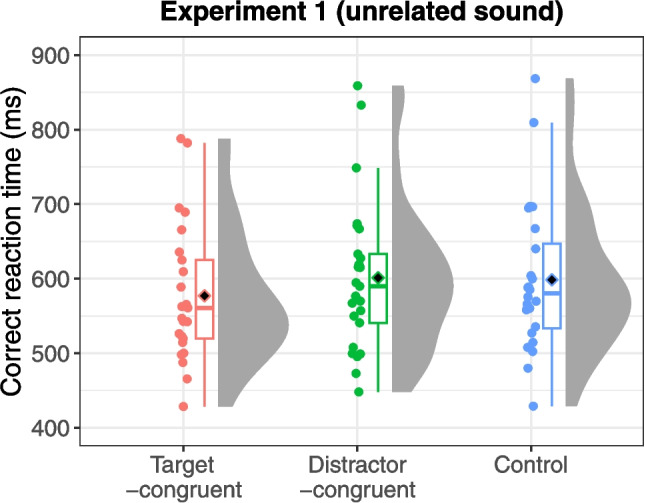
Reaction times across sound conditions in Experiment 1 with unrelated sound as control. Black diamonds on the box plots represent overall mean values, and each dot represents the mean reaction time for individual participants

Error trials (4.45% of all trials) and outliers (0.61% of correct trials) were excluded from the analysis. Figure 3 presents the mean reaction times for each sound condition: target-congruent, distractor-congruent, and “unrelated sound” control. The ANOVA revealed a significant main effect of sound condition, *F*(2,48) = 12.03, *p* < 0.001, η_G_^2^ = 0.019, ɛ = 0.943. Post hoc tests indicated that reaction times were significantly shorter in the target-congruent condition compared with both the control condition, *t*(24) = 4.28, *p* < 0.001, and the distractor-congruent condition, *t*(24) = 3.50, *p* = 0.004. However, no significant difference was found in reaction time between the distractor-congruent and control conditions, *t*(24) = 1.52, *p* = 0.142. These findings indicate that characteristic sounds facilitate visual search for dynamic action scenes without causing interference.

#### Experiment 2 (white noise)

**Fig. 4:**
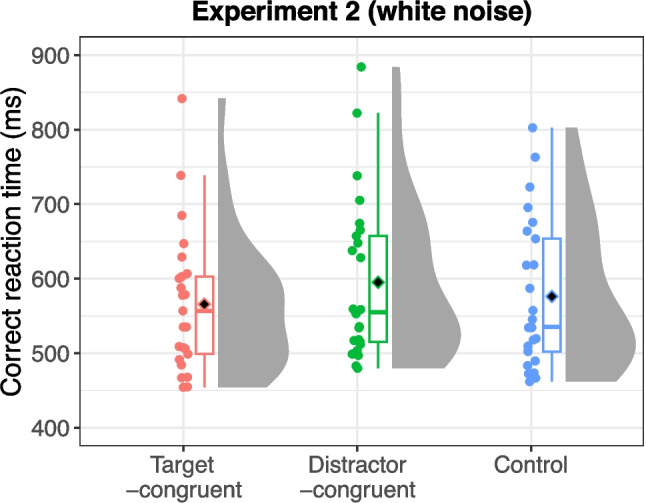
Reaction times for each sound condition in Experiment 2 (with white noise as the control). Black diamonds on the box plots represent overall mean values, and each dot represents the mean reaction time for individual participants

Error trials (3.65% of all trials) and outliers (0.55% of correct trials) were excluded from the analysis. Figure 4 presents the mean reaction times for each sound condition: target-congruent, distractor-congruent, and “white-noise” control. An ANOVA revealed a significant main effect of sound condition, *F*(2,48) = 7.99, *p* = 0.001, η_G_^2^ = 0.015, ɛ = 0.990. Post hoc tests indicated that reaction times were not significantly shorter in the target-congruent condition compared with the control condition, *t*(24) = 1.41, *p* = 0.170. Interestingly, however, reaction times were significantly longer in the distractor-congruent condition compared with both the target-congruent condition, *t*(24) = 3.77, *p* = 0.003, and the control condition, *t*(24) = 2.65, *p* = 0.028. These findings contrast with those of Experiment 1, indicating that characteristic sounds interfered with the visual search for dynamic action scenes.

#### Experiment 3 (no sound)

**Fig. 5:**
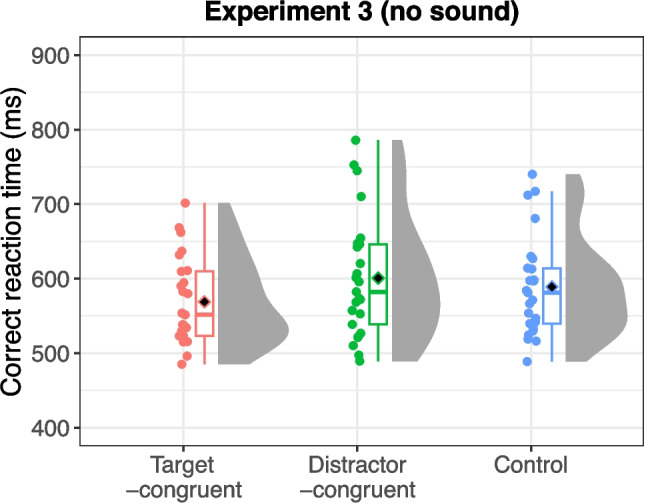
Reaction times for each sound condition in Experiment 3 (with no sound as the control). Black diamonds on the box plots represent overall mean values, and each dot indicates the mean reaction time for individual participants

Error trials (3.80% of all trials) and outliers (1.06% of correct trials) were excluded from the analysis. Figure 5 presents the mean reaction times for each sound condition: target-congruent, distractor-congruent, and “no sound” control. An ANOVA revealed a significant main effect of sound condition, *F*(2,48) = 13.90, *p* < 0.001, η_G_^2^ = 0.036, ɛ = 0.876. Post hoc tests indicated that reaction times were significantly shorter in the target-congruent condition compared with both the control condition, *t*(24) = 4.11, *p* < 0.001, and the distractor-congruent condition, *t*(24) = 4.66, *p* < 0.001. However, there was no significant difference, although some trends were observed, in reaction time between the distractor-congruent condition and the control condition, *t*(24) = 1.84, *p* = 0.078. These findings indicate that characteristic sounds facilitated the visual search without causing interference.

#### Comparison with the search from Maezawa et al. (2022)

We conducted action-scene search tasks separately using dynamic images (video clips) with three distinct control sounds. The present results, with the exception of Experiment 2, generally replicated the auditory facilitation of search performance observed in previous studies (Iordanescu et al., 2008, 2010, 2011; Kvasova et al., 2019; Maezawa et al., 2022). This finding is unsurprising, as the facilitation effects of target-congruent sounds have consistently been found not only in search tasks but also in identification studies (Chen & Spence, 2010, 2011; Cox & Hong, 2015; Hein et al., 2007; Mastroberardino et al., 2015; Molholm et al., 2004; Spilcke-Liss et al., 2019; Zweig et al., 2015). However, we did not observe consistent interference across the experiments: only one of the three experiments (Experiment 2) demonstrated auditory interference. These weak effects of the distractor-congruent sound contrast with the trend observed in the search paradigm using static action-scene images (Maezawa et al., 2022), where interference was generally noted in five of six experiments.

To compare the effect sizes of the distractor-congruent sound in the present study with those in the previous study, we analyzed reaction time data from experiments that employed the same control conditions (Experiments 4–6 of Maezawa et al., 2022). The Cohen’s *d* values represent the standardized differences in reaction times between the distractor-congruent condition and control conditions. Table 1 presents these values across different search paradigms, highlighting the discrepancies in interference effects. The comparison of effect sizes revealed a consistent trend across studies. In particular, the smallest values occurred with the “unrelated” control sound, followed by “no sound” and “white noise.” The overall effect sizes in the present study were smaller than those in Maezawa et al. (2022), reinforcing the observation that interference effects were relatively weak in the current search task.

**Table 1:** Effect sizes of interference across different search paradigms

Control sound	Dynamic action-scene search (present study)			Static action-scene search (Maezawa et al., 2022)		
	*N*	*p* value	Cohen's *d*	*N*	*p* value	Cohen's *d*
Unrelated	25	0.142	0.095	25	0.039	0.133
White noise	25	0.028	0.190	25	< 0.001	0.500
No sound	25	0.078	0.171	25	< 0.001	0.418

To further investigate the weak auditory interference, we analyzed error responses in target localization under the distractor-congruent condition, where a nontarget item related to the auditory cue (i.e., a distractor) was included in the search display (see Fig. 2). Two additional nontarget items were also present in the display. If interference occurred, we would expect a higher rate of localization errors directed toward the distractor item rather than the nontargets. Based on this hypothesis, we assessed whether the rate of error localization responses exceeded the chance level of 33.33% (one of three nontarget items) at the distractor location. Table 2 compares the error response rates between the present and previous studies (Experiments 4–6 of Maezawa et al., 2022). Our findings indicate a minimal response bias toward the distractor item (one-way bimodal test: *p* = 0.114 in Experiment 1; *p* = 0.164 in Experiment 2; and *p* = 0.180 in Experiment 3). This contrasts with the significant bias observed in previous research and further supports the conclusion that interference effects were relatively weak in the present study.

**Table 2:** Percentage of error responses according to distractor location

Control sound	Dynamic action-scene search (present study)					Static action-scene search (Maezawa et al., 2022)				
	Distractor	%	Two fillers	%	*p* value	Distractor	%	Two fillers	%	*p* value
Unrelated	37	39.78%	56	60.22%	0.114	58	44.96%	71	55.04%	0.004
White noise	32	39.02%	50	60.98%	0.164	62	45.93%	73	54.07%	002
No sound	36	38.30%	58	61.70%	0.180	52	49.52%	53	50.48%	< 0.001

## Discussion

The findings from Experiments 1–3 did not fully replicate the interference effects reported by Maezawa et al. (2022). Instead, they align more closely with prior studies, indicating no significant interference during visual searches (Iordanescu et al., 2008, 2010, 2011; Kvasova et al., 2019). Notably, however, these results do not establish that visual search inherently involves mechanisms for filtering irrelevant auditory or visual information. Furthermore, Experiment 2 provides compelling evidence of the influence of distractor-congruent sounds that cannot be overlooked (see Table 1). Thus, rather than concluding in binary terms (i.e., interference is present or absent), it seems more appropriate to consider that interference effects may vary according to specific conditions or task demands.

Our updated perspective, based on the results presented in Table 1, suggests that the size of interference effects varies depending on the type of control sound employed. One explanation is that certain control sounds may increase baseline reaction times due to their acoustic properties, leading to an underestimation of the response delays caused by distractor-congruent sounds. As Maezawa et al. (2022) discussed, this baseline inflation could occur when “unrelated sounds” unintentionally enhance visual representations that are semantically distinct from the target. As a result, baseline reaction times are prolonged, and interference could appear smaller compared with searches with nonsemantic controls such as “white noise” or “no sound.” Moreover, in searches with the “no sound” condition, delays may arise from a lack of auditory cues signaling the onset of the visual stimulus, reducing alertness and further inflating baseline reaction times. This would also diminish the observed interference effects. Considering these factors, searches with the “white noise” control seem to offer the most accurate reflection of interference effects, as seen in our results. Nevertheless, some issues remain unresolved. Although white noise is acoustically neutral relative to more characteristic sounds, it is still unnatural, and our experiments did not account for variation in the sound pressure level of auditory stimuli. While unlikely, this could still introduce confounding variables into the reaction time analysis.

To address these concerns, we modified the control conditions in Experiments 4 and 5 by replacing white noise with a mixture of action-related characteristic sounds to provide a more valid acoustic control. Experiment 4 employed dynamic images as search stimuli, whereas Experiment 5 focused on static images, following the methodology of Maezawa et al. (2022). These adjustments were made to rigorously reassess the interference effects observed when using white noise as the control in both the current and previous approaches, particularly in scenarios where specific acoustic properties were less likely to distort the findings.

## Experiments 4 and 5

Experiments 4 and 5 replicated the effects of sound presentations on visual searches involving dynamic (Experiment 4) and static (Experiment 5) action-scene images. The search tasks were identical to those in the prior experiments, with the primary modification being the introduction of a mixture of characteristic sounds in place of white noise. This change sought to further investigate the conditions under which interference occurs and to eliminate potential confounding factors related to specific acoustic properties. In addition, these studies compared the sizes of interference effects across different approaches (dynamic vs. static action-scene searches), with particular attention given to the interaction between interference effects and the types of search tasks.

### Methods

#### Participants

A new group of 50 naïve undergraduate and graduate students from Hokkaido University participated in Experiments 4 and 5 (22 men and 28 women; mean age = 19.98 years, range: 18–28 years). They were evenly assigned to each experiment. All participants had normal color vision, normal hearing ability, and normal or corrected-to-normal visual acuity.

#### Apparatus, stimuli, and procedure

**Fig. 6:**
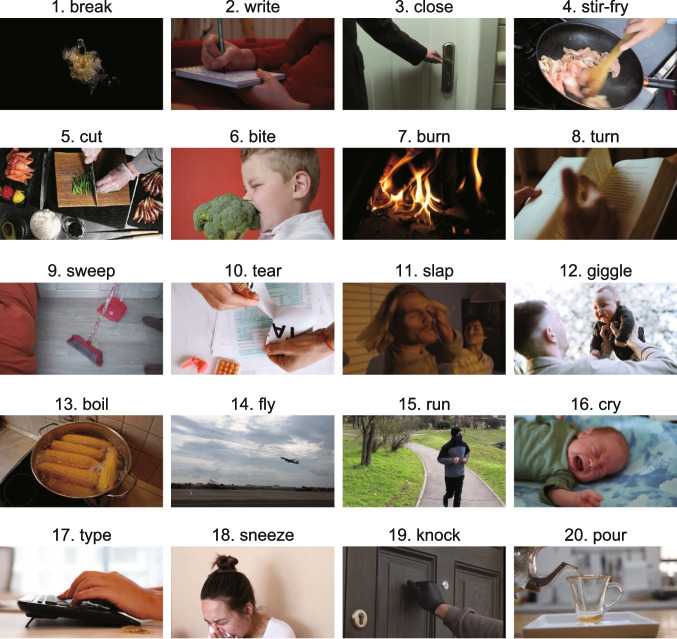
Representative examples of the static action-scene images used in Experiment 5

In Experiment 4, the dynamic images (video clips) from Experiments 1–3 were reused as search items, whereas Experiment 5 utilized 20 static images (color pictures) derived from these video clips (Fig. 6). These static images were captured at a nearby moment when an event corresponding to the action target name (e.g., “break”) occurred in the video. Both experiments implemented a “mixed sound” control instead of a “white noise” control. The mixed sounds were generated by combining 16 action-related sounds for each trial, excluding sounds associated with the visual items in the search display. This approach ensured that the control sound contained no semantic information related to the target or distractor scenes. To maintain consistent sound pressure levels across various sound conditions (i.e., target-congruent, distractor-congruent, and control), the amplitudes of the auditory stimuli were adjusted according to the root mean square value. Except for adjustments in visual stimuli and control sound, the task procedures were consistent with those of the previous experiments.

### Results

#### Experiment 4 (dynamic action-scene search)

**Fig. 7:**
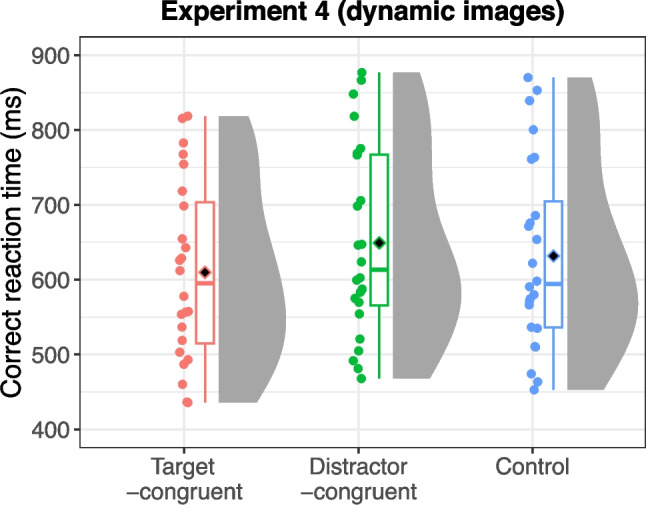
Reaction times for each sound condition in Experiment 4 (with dynamic images and the mixed sound control). Black diamonds on the box plots represent overall mean values, and each dot represents the mean reaction time for individual participants

Error trials (3.17% of all trials) and outliers (0.83% of correct trials) were excluded from the analysis. Figure 7 presents the mean reaction times for each sound condition: target-congruent, distractor-congruent, and “mixed sound” control. An ANOVA revealed a significant main effect of sound condition, *F*(2,48) = 26.27, *p* < 0.001, η_G_^2^ = 0.015, ɛ = 0.969. Post hoc tests indicated that reaction times were significantly shorter in the target-congruent condition compared with both the control condition, *t*(24) = 3.95, *p* = 0.001, and the distractor-congruent condition, *t*(24) = 6.68, *p* < 0.001. Conversely, reaction times were significantly longer in the distractor-congruent condition than in the control condition, *t*(24) = 3.64, *p* = 0.001. These findings indicate that characteristic sounds both facilitated and interfered with visual searches for dynamic action scenes.

#### Experiment 5 (static action-scene search)

**Fig. 8:**
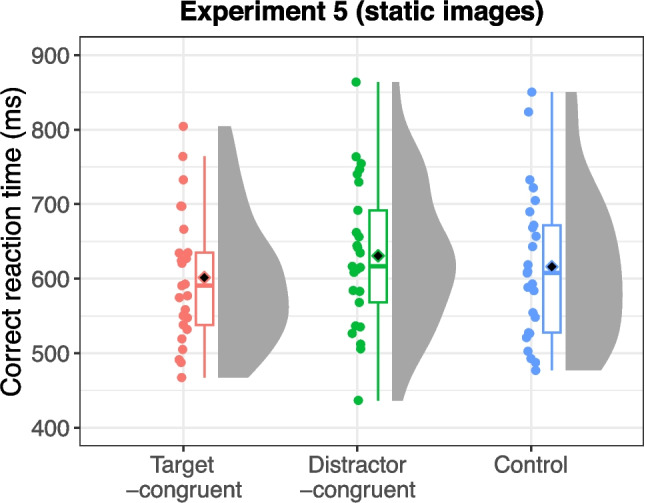
Reaction times for each sound condition in Experiment 5 (with static images and the mixed sound control). Black diamonds on the box plots represent overall mean values, and each dot indicates the mean reaction time for individual participants

Error trials (6.08% of all trials) and outliers (1.22% of correct trials) were excluded from the analysis. Figure 8 presents the mean reaction times for each sound condition. The ANOVA revealed a significant main effect of sound condition, *F*(2,48) = 9.03, *p* < 0.001, η_G_^2^ = 0.016, ɛ = 0.969. Post hoc tests indicated that reaction times were significantly shorter in the target-congruent condition than in the distractor-congruent condition, *t*(24) = 4.65, *p* < 0.001. Conversely, there were no significant differences in reaction times, although some trends were observed, between the target-congruent and control conditions, *t*(24) = 2.12, *p* = 0.089, and between the distractor-congruent and control conditions, *t*(24) = 1.98, *p* = 0.089.

The post hoc tests were likely affected by reduced statistical power due to multiple comparisons. The effect sizes for both facilitation and interference (*d* = 0.153 for both) were similar to those observed in Experiment 4 (*d* values = 0.155 and 0.144, respectively). Furthermore, the difference in reaction times between the target-congruent and distractor-congruent conditions suggests that, compared with the control condition, responses were faster in the target-congruent condition (facilitation) and slower in the distractor-congruent condition (interference). To further explore this trend, particularly the interaction between interference and the experimental approach, additional analyses were conducted.

#### Comparison between experiments 4 and 5

**Fig. 9:**
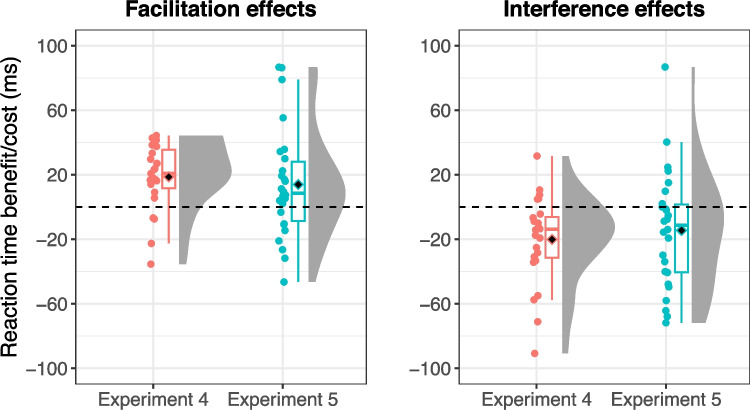
Reaction time benefits and costs relative to the baseline. Black diamonds on the box plots represent overall mean values, and each dot indicates the mean reaction time for individual participants

The reaction time benefits and costs for the target-congruent and distractor-congruent conditions were calculated relative to the control condition, with positive values indicating facilitation and negative values indicating interference (Fig. 9). Each value was compared among experiments using pairwise *t* tests with Holm correction. The results revealed no significant differences in the magnitude of facilitation between Experiment 4 (dynamic action-scene search) and Experiment 5 (static action-scene search), *t*(48) = 0.74, *p* = 0.926, *d* = 0.209, or in the magnitude of interference between the experiments, *t*(48) = 0.54, *p* = 0.595, *d* = 0.151. These findings suggest that the sizes of facilitation and interference effects did not depend on the types of search tasks (i.e., dynamic or static action-scene searches).

### Discussion

The results of Experiments 4 and 5 demonstrated both auditory facilitation and interference during visual search tasks, regardless of the type of search items used. These findings align with previous experiments that employed “white noise” as a control, confirming the presence of interference effects in visual search. These findings are in disagreement with the hypothesis that interference effects are inherently absent because of a goal-directed attentional set (Iordanescu et al., 2008, 2010, 2011).

However, interference fluctuates based on experimental conditions and task requirements (see Table 1). A potential factor influencing this variability is the type of control sound used. In fact, experiments employing “unrelated sound” or “no sound” controls tend to exhibit relatively reduced interference, further supporting this variability. Interestingly, using dynamic action-scene images (as in Experiments 1–3) resulted in smaller interference effects compared with static action-scene searches in the previous study by Maezawa et al. (2022). Notably, these trends were not replicated in the comparison between Experiments 4 and 5, as seen by the effect sizes (*d* values = 0.153 and 0.144, respectively) that were smaller than those observed in “white noise” experiments (*d* values = 0.190 and 0.500). Accordingly, although a comparison of effect sizes alone makes it challenging to explain the variability in interference, the current study demonstrated a noteworthy pattern for consideration.

## General discussion

This study explored the influence of semantic associations between auditory and visual stimuli on action-scene searches (Maezawa et al., 2022), utilizing dynamic video clips as the search stimuli. Participants were exposed to characteristic sounds related to either target or nontarget action scenes at the start of their searches. Expectedly, the three experiments demonstrated auditory facilitation effects across various control sound. However, interference effects were weaker and only emerged when white noise was used as the control sound (Experiment 2). Furthermore, the interference effect sizes were smaller than those reported by Maezawa et al. (2022). To investigate further, control experiments (4 and 5) were conducted, successfully replicating interference and excluding the possibility that the findings in Experiment 2 were coincidental. Overall, these results confirm the presence of auditory interference, although the effects may be relatively weak under certain conditions.

The current observations regarding auditory interference (Experiments 2 and 4), although weak, challenge the conventional notion that distractor-related semantic information can be ignored in search tasks due to a goal-directed attentional set (Iordanescu et al., 2008). Our results are somewhat exceptional. In most cases, observers rely on prior knowledge of target objects, allowing them to focus on easily recognizable visual features (e.g., four legs and a tail for dogs; Chen & Spence, 2010). By maintaining these mental representations during the search process, individuals can purposefully prioritize such information and effectively filter out irrelevant distractors, even those with a high stimulus intensity (Chen & Zelinsky, 2006). This view (see also Gaspelin & Luck, 2018a, b; Reeder & Peelen, 2013; Theeuwes, 2018) is supported by numerous studies that report no interference in crossmodal searches, particularly when real objects are the targets (Iordanescu et al., 2008, 2010, 2011; Kvasova et al., 2019). Therefore, instead of rejecting the established view, we propose that auditory facilitation coexist with interference effects.

Moreover, it is essential to understand that the variability in interference effects, particularly regarding the occurrence of interference, can be interpreted through the framework of goal-directed search mechanisms outlined previously. A crucial aspect of this interpretation is the lack or reduction of target designation during the search process (Chen & Zelinsky, 2006). When prior knowledge about the target, including any previews, is unavailable, the ability to prioritize may be compromised, leading to an increased influence from distractor items. In other words, when more information about the target is available from prior knowledge, the likelihood of interference is reduced.

This mechanism can potentially explain the differences in interference patterns between this and previous studies involving the action-scene search task (Maezawa et al., 2022), as seen in Table 1. The video stimuli utilized in the present study incorporate dynamic features commonly associated with action, which makes them more likely to be encountered in real-life scenarios than static snapshots (Maezawa et al., 2022). This implies that individuals may be more capable of associating prior knowledge with dynamic targets, resulting in a relatively easier target designation compared with static images. Indeed, except for certain comparisons such as Experiments 4 and 5, our study showed weak interference, demonstrating a trend toward the patterns observed in searches involving real objects (although not entirely identical; Iordanescu et al., 2008, 2010, 2011; Kvasova et al., 2019). However, this study did not evaluate whether participants possessed adequate prior knowledge, necessitating further research that manipulates factors such as the availability of previews or prior experiences with audiovisual stimuli to confirm this hypothesis.

The influence of prior knowledge on search outcomes is further supported by additional evidence. Repeated exposure to co-occurring visual and auditory stimuli can enhance the conceptual association between auditory cues and visual targets, potentially modulating the impact of characteristic sounds (e.g., Zweig et al., 2015). Studies showing facilitation and interference effects in identification or search tasks typically focus on standard audiovisual pairings that are likely acquired through everyday experiences (e.g., Smith et al., 2007), with minimal exploration of alternative combinations. However, disruptions in the conventional associations between these stimuli can notably reduce these auditory effects (Iordanescu et al., 2008, 2011). This phenomenon was partially illustrated by the word-name search task (Iordanescu et al., 2011), where real objects were substituted with words representing their names. In this experiment, facilitation was observed only when spoken names accompanied the words, but not when characteristic sounds were used. This distinction likely results from the consistent paring of written words with spoken names, whereas such words are infrequently associated with characteristic sounds such as barking.

Taken together, the effects of characteristic sounds observed in this study may arise from interactions rooted in higher-order semantic representations, a widely accepted perspective (e.g., Chen & Spence, 2010; Potter, 1999). Nevertheless, it is crucial to explore the possibility that low-level auditory and visual features influence the search outcomes. In particular, the auditory and visual stimuli used in our paradigm were not synchronized in time; the onset of sounds and visual events was largely asynchronous. This suggests the possibility that stimulus pairs with smaller temporal differences could have had a more significant impact, potentially intensifying facilitation or interference effects compared with those with greater temporal discrepancies. However, this explanation seems less pertinent in the context of our findings. Our supplementary comparisons of reaction times under target-congruent conditions (using the data from control Experiment 4) revealed faster responses even for stimuli such as “stir-fry,” “burn,” and “fly,” where the temporal alignment between auditory and visual events was likely of lesser importance. These results imply that low-level features alone cannot fully account for the observed effects, reinforcing the assertion that higher-order cognitive processes play a substantial role.

In summary, our results highlight the presence and variability of interference in crossmodal search tasks, particularly in relation to the characteristics of search processes, in line with our prior findings (Maezawa et al., 2022). These results do not necessarily dispute the established perspective on search mechanisms, wherein observers often ignore distractor information during searches (Iordanescu et al., 2008, 2010, 2011; Kvasova et al., 2019). This may be because a goal-directed strategy is commonly employed (Chen & Zelinsky, 2006). However, the influence of distractors may be amplified based on the strength of target designation. Considering that the present study did not explore this factor in detail, further studies are needed to investigate this aspect and determine the underlying factors contributing to interference.

## Data Availability

The data and significant program code will be made available after acceptance via the Open Science Framework (https://osf.io/kgzqd/), and none of the experiments was preregistered.
